# Abundance of impacted forest patches less than 5 km^2^ is a key driver of the incidence of malaria in Amazonian Brazil

**DOI:** 10.1038/s41598-018-25344-5

**Published:** 2018-05-04

**Authors:** Leonardo Suveges Moreira Chaves, Jan E. Conn, Rossana Verónica Mendoza López, Maria Anice Mureb Sallum

**Affiliations:** 1 Departamento de Epidemiologia, Faculdade de Saúde Pública, Universidade de São Paulo, São Paulo, SP Brazil; 2 Department of Biomedical Sciences, School of Public Health, University at Albany (State University of New York), Albany, NY USA; 3 Wadsworth Center, New York State Department of Health, Albany, NY USA; 4 Center of Translational Investigation in Oncology, Cancer Institute of the State of São Paulo, São Paulo, SP Brazil

## Abstract

The precise role that deforestation for agricultural settlements and commercial forest products plays in promoting or inhibiting malaria incidence in Amazonian Brazil is controversial. Using publically available databases, we analyzed temporal malaria incidence (2009–2015) in municipalities of nine Amazonian states in relation to ecologically defined variables: (i) deforestation (rate of forest clearing over time); (ii) degraded forest (degree of human disturbance and openness of forest canopy for logging) and (iii) impacted forest (sum of deforested and degraded forest patches). We found that areas affected by one kilometer square of deforestation produced 27 new malaria cases (r² = 0.78; F1,10 = 35.81; P < 0.001). Unexpectedly, we found both a highly significant positive correlation between number of impacted forest patches less than 5 km^2^ and malaria cases, and that these patch sizes accounted for greater than ~95% of all patches in the study area. There was a significantly negative correlation between extraction forestry economic indices and malaria cases. Our results emphasize not only that deforestation promotes malaria incidence, but also that it directly or indirectly results in a low Human Development Index, and favors environmental conditions that promote malaria vector proliferation.

## Introduction

The relationship between deforestation and malaria in the Amazon is controversial, with some studies claiming that deforestation can diminish malaria incidence^1,2^, and others that *Plasmodium* Marchiafava & Celli transmission risk is enhanced by deforestation^3–6^. An extensive recent review found no overwhelming evidence for a direct, consistent relationship between deforestation, forests, and malaria incidence^7^, but results of the surveyed literature showed that contexts, specific questions/hypotheses, and methodologies employed differed, and strongly influenced outcomes. The Amazon biome is the largest extant area of continuous tropical rain forest, and one of the last remaining active forest frontiers in the world^8^. Exploitation and export of natural resources, including mahogany and other wood of high commercial value worldwide, are linked directly and indirectly to Brazilian economic growth, and associated with the development of new agriculture frontiers^9^. Continuous demands for economic, social and political transformation^10^ lead to increased forest destruction, changes in land cover, ecosystem degradation, and reduced biodiversity^11–13^. Furthermore, illegal logging is widespread, and usually associated with precarious human living conditions^14^. Rural development policies in the Amazon are primarily focused on land distribution, allegedly to eliminate extreme poverty and decrease social inequalities. Nevertheless, both small and large landholders play an important role in deforestation for the development of pastures and crops^15,16^. Economic growth in Brazil is partially dependent on the availability of new forest areas for agri-business investment and human occupation, hastening destruction of the Amazon forest^17,18^. According to data from the Brazilian Institute of Geography and Statistics (IBGE)^19^, the inclusion of forestry products in the commercial exploitation of natural resources in 2015 represented an increase of approximately USD$ 1.5 billion in the gross value of Brazilian commodities. Commercial forest products included approximately 26 million tons of firewood, 12 million tons of logs and 331 thousand tons of wood charcoal. Could there be an unexplored, possibly indirect association between forestry product production and malaria incidence?

Malaria in the Amazon is characterized by a low transmission landscape, interspersed with malaria hotspots, that are spatially and temporally variable^20,21^. In such a heterogeneous landscape, malaria is a persistent public health threat^1,22–24^. It is also seasonal, and has been shown to be linked to rainfall, dry season length and/or survival of the main malaria vector *Nyssorhynchus darlingi* (Root)^25–27^. The risk of acquiring malaria in an agricultural settlement is mainly associated with environmental and socioeconomic determinants that include characteristics of human dwellings, proximity of dwellings to larval habitats of *Ny. darlingi*, exposure to infected biting females, and forest cover^21,23,26,28,29^; access to health facilities, diagnostic and treatment for malaria, the educational level of human communities that is associated with community organization and knowledge relative to malaria transmission, history of previous migration, and the economic development of the communities, among others^29–31^. Moreover, economic growth of an Amazonian rural settlement depends on the development of a market network, robust transport system and social and public infrastructure, including construction of health facilities. These activities are associated with what is defined as pendulum mobility^32^, which implies continuous human movement between rural areas and cities, a process that can establish and sustain a high incidence of malaria^31^.

Deforestation and changes in land use or land cover are frequently associated with the emergence of larval habitats suitable for *Ny*. *darlingi*, i.e., small streams or igarapés with partial shade, usually located at the forest edge. The size of a forest patch can increase the distance between larval habitat and forest edge, and modify sunlight incidence, increasing *Ny. darlingi* abundance^33–36^. Kweka *et al*.^37^ found that land cover change is a key driver for increasing the abundance of mosquitoes that coincidentally are vectors of pathogens. Although there is evidence that links deforestation and malaria occurrence^4,38^ a statistical analysis of deforested patch sizes, and the potential impact of commercial forestry products on malaria incidence are missing, and we hypothesize that they are overlooked economic drivers in the Amazonian ecosystem with important consequences for *Plasmodium* transmission. The objectives of this study were to: (1) assess the impact of deforestation (km²) on malaria; (2) address temporal and spatial correlation of malaria and deforestation; and (3) verify association between selective logging, charcoal production and malaria; (4) verify correlation of rainfall and the number of malaria cases, deforestation and degraded forest; and between the municipalities with the highest number of malaria cases; (5) test the impact of exploitation of timber and charcoal on malaria incidence, and (6) address the Amazon social service chain of connectivity (supplies, transport, trade and public service demands) and malaria incidence across these network linkages.

## Materials and Methods

### Database

**Figure 1:**
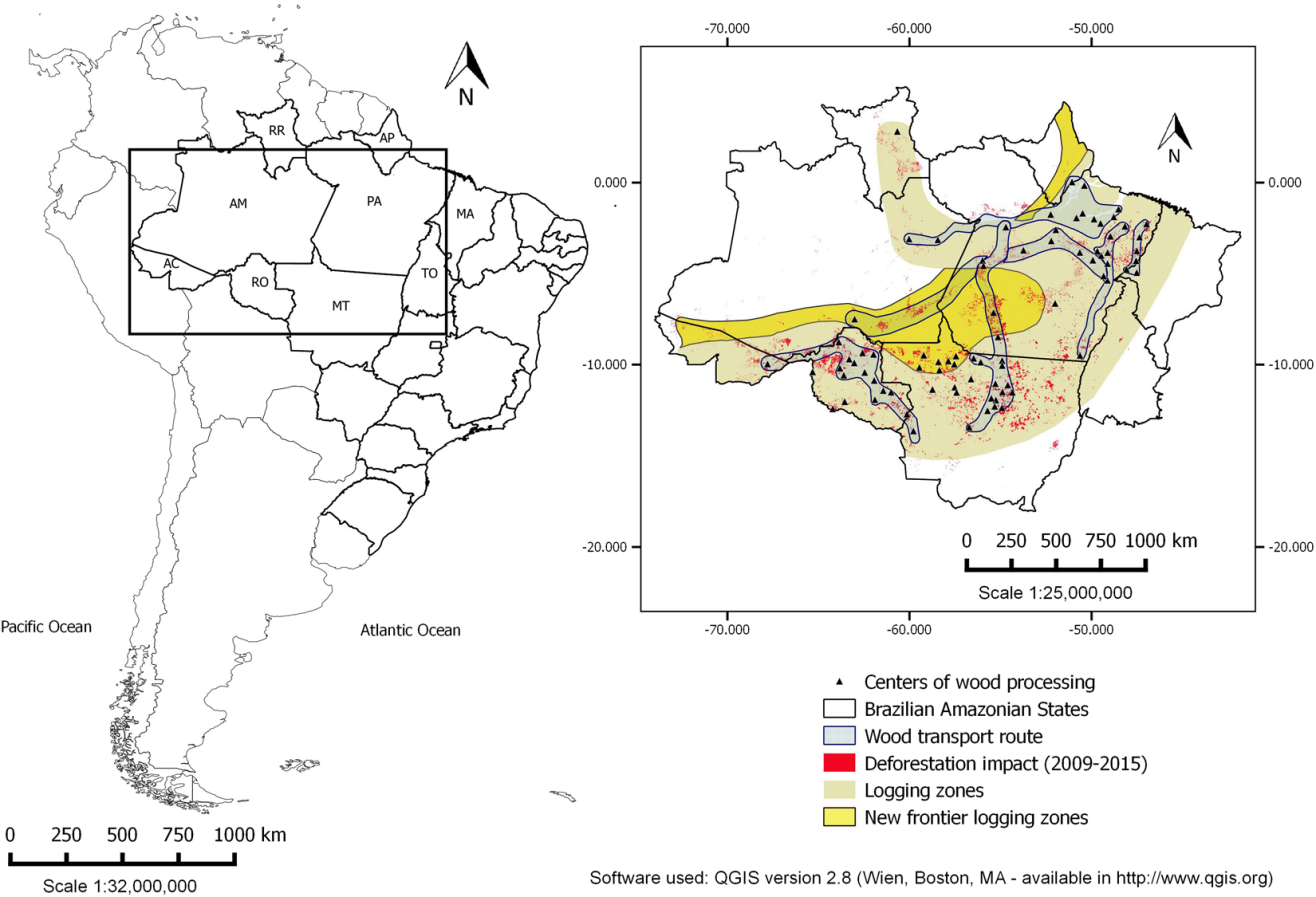
Map of the geographical area studied. Highlighted: Brazilian Amazonian states and the areas of timber distribution, deforestation/degradation, and connectivity between regions of forest exploitation and transport, commercial regions of established forestry production and new frontiers of exploitation.

Malaria incidence, forest degradation and deforestation data used in the analysis included all municipalities of the endemic states of Acre, Amapá, Amazonas, Maranhão, Mato Grosso, Pará, Rondônia, Roraima and Tocantins (Fig. 1) from 2009–2015. The Amazon biome represents the spatial unit, and months from 2009–2015, the temporal unit for the variables. The premise for wood transport routes is the presence of the roads and the new frontier logging zones are classified according to the forest type (commercial tree species) and conditions of access (Fig. 1). Asner *et al*.^39^ verified that nearly all logging occurs within 25 km of main roads, and within that area, the probability of deforestation for a logged forest (algorithm provided estimates of canopy damage) was up to four times greater than for unlogged forests, helping to support the new frontier logging zone depicted in Fig. 1.

Deforestation and degradation data were obtained as free vectorial shapefiles from the Imazon^©^ (Institute of Man and Environment of the Amazon) web site (http://www.imazongeo.org.br) that created a system called Deforestation Alert System (DAS). Autochthonous cases of malaria in the Amazonian region were obtained from the Ministry of Health platform (http://portalsaude.saude.gov.br), this data refer to total of malaria cases caused by both *Plasmodium vivax* Grassi & Feletti (responsible for most cases) and *Plasmodium falciparum* Welch (less frequent). Data of the network of the geopolitical and social links, and of the geopolitical centers and managerial importance of the territory were obtained from the Brazilian Institute of Geography and Statistics (Região de influência das cidades, 2007 – IBGE (http://www.ibge.gov.br)). The criteria to construct the links between the endemic municipalities of the states were based on the number of autochthonous malaria cases. Thus, the municipalities that did not have reached 80% of total annual malaria case participation, whose annual malaria cases totaled less than 20,000 in that state, were excluded from the geopolitical map, i.e., municipalities of the states of Roraima, Mato Grosso, Maranhão and Tocantins.

Accumulated rainfall (mm³) was used to determine correlation between malaria cases and deforestation. Rainfall data were obtained from automatic meteorological stations located in the areas included in this study and available on the Brazilian Institute of Meteorology (Instituto Nacional de Meteorologia – INMET (http://www.inmet.gov.br)). Data of economic variables, timber (logs and firewood) and wood charcoal, were obtained from the Brazilian Institute of Geography and Statistics databases (Instituto Brasileiro de Geografia e Estatística – IBGE (http://www.ibge.gov.br)).

### The Imazon^©^ Deforestation Alert System (DAS)

The Imazon DAS is based on data of the detection of deforestation in real time of the DETER (Real Time Deforestation Detect) and PRODES (Deforestation Monitoring Project in the Amazon). DETER and PRODES provide data that are collected and administrated by the Brazilian Institute for Space Research – INPE (http://www.obt.inpe.br/prodes/). DETER uses satellite images supplied by MODIS (NASA) (MOD09GQ and MOD09GA), with cloud filtration, to monitor the forest and guide the Brazilian Institute of Environment and Renewable Natural Resources (IBAMA) to inspect illegal deforestation. The detection capacity of Imazon DAS is 0.05 km² (with 100% accuracy and 78% correlation for polygons 0.03–1.00 km² compared with Landsat images)^40^. Imazon images allow quantifiable estimation of small deforested areas by MODIS image composites^40,41^. As our study verified the temporal correlation of deforestation and malaria over time, we adopted the Imazon DAS for our analysis, because this system is more systematic and samples are taken more frequently compared with Landsat images^42,43^.

### Ecological concepts and economic variables

We adopted the definitions of deforestation and degradation in the text box from a study by Lima *et al*.^7^ For our analysis, impacted forest patches were quantified using the sum of the deforested and of the degraded areas in square kilometers. The number and the size of patches in square kilometers were adopted based on bionomics characteristics of *Ny. darlingi* in certain landscapes, because this species uses the forest edge for maintenance of its immature forms^44^.

The economic variables were related to exploitation of forest natural resources, i.e., the scale of production and the value of the products such as logs, firewood and charcoal. The rationale for selecting these variables is that they have a direct impact on deforestation activities. In contrast, variables such as cattle ranching are only indirectly correlated with deforestation^45^, and not were measured here. To convert the value of production from Brazilian currency (Real) to USD$ (United States Dollar), we adopted the daily mean exchange rate from the period between the years 2009–2015.

### Data analysis

The total area of deforestation, degradation, and both and the number of malaria cases were calculated monthly. For statistical descriptive analyses we employed boxplots and barplot graphs to determine whether there were an association between malaria and deforested, forest degraded and both (impacted forest) area. Impacted forest areas were employed to construct polygons, thus the patches were used to determine the association between the number of malaria cases and patterns of deforestation. Six categories of patch sizes were defined: number of patches larger than 5 km², and number of patches smaller than 5 km², 0.5 km², 0.25 km², 0.10 km² and 0.08 km². To evaluate the percentage of the annual decrease of the number of impacted forest patches we included one more category that was defined as the number of patches smaller than 15 km² in each month.

Spearman’s correlation index was utilized to verify the correlation among rainfall, impacted forest, factors of the economic activity (forestry timber production) and the number of malaria cases. The non-parametric test of Kolmogorov-Smirnov was employed to verify whether the number of malaria cases had a normal distribution (at a significance level of 5%). Quarterly moving means of deforestation impact (km²) and accumulated rainfall (mm³) were employed to verify an exploratory correlation of the seasonality on the number of malaria cases and the impact of deforestation (km²). Models of simple and multiple linear regression were adopted for the six categories of impacted forest patch sizes in km², abundance of impacted forest patch units, rainfall (mm³) and number of malaria cases. Correlation between economic variables (production and aggregate value) and the number of malaria cases was evaluated using the ratio between the amount of money earned in USD$ and the total production (m³) of forestry extraction in the Brazilian Amazon, and analyzed by Pearson’s statistical test. We employed the software QGIS 2.8 (Wien, Boston, MA) for mapping, analysis and quantification of the geographical polygon data. The statistical analyses were carried out using R v.2.15.3 software (R Development Core Team, R Foundation for Statistical Computing, Austria) R-project (available at http://www.r-project.org) and stats package.

## Results

From 2009–2015, malaria case numbers in Amazonian Brazil varied from a high of 327,142 in 2010 to a low of 138,697 in 2015. Overall, during the study period, 32,698 km² of forest were impacted by deforestation and forest degradation. The total area was distributed into 36,689 polygons with areas that ranged from less than 0.05 km² to 180 km².

Table 1 shows a correlation matrix where the unit of analysis was the size of the deforested patches. Here, we sum the number of the satellite polygons which represented the same patches sizes, with or without forest degradation, in a landscape. There is a high positive correlation between the number of impacted patches from 5 km² to 0.25 km² and number of malaria cases. When added to the number of patches of less than 0.10 km², however, this correlation loses statistical significance (r = 0.46; *P* = 0.12).

**Table 1:** Matrix of the correlation between the variables of deforestation activities, landscape and the environment (rainfall), and the number of malaria cases in Brazilian Amazonian States.

Variables	No. of malaria cases	Deforestation (km²)	Degradation (km²)	Impact (km²)	Accum. rainfall (mm³)	No. of patches (>5 km²)	No. of patches (<5 km²)	No. of patches (<0.5 km²)	No. of patches (<0.25 km²)	No. of patches (<0.10 km²)
Deforestation (km²)	0.82^c^									
Degradation (km²)	0.58	0.87^b^								
Impact (km²)	0.73^d^	0.96^a^	0.93^a^							
Accum. rainfall (mm³)	−0.80^c^	−0.90^a^	−0.82^b^	−0.86^b^						
No. of patches (>5 km²)	0.24	0.63^e^	0.87^b^	0.68^d^	−0.60^e^					
No. of patches (<5 km²)	0.81^c^	0.96^a^	0.87^a^	0.94^a^	−0.94^a^	0.64^e^				
No. of patches (<0.5 km²)	0.80^c^	0.89^a^	0.83^b^	0.89^a^	−0.98^a^	0.56	0.96^a^			
No. of patches (< 0.25 km²)	0.72^d^	0.84^b^	0.82^b^	0.85^b^	−0.94^a^	0.55	0.88^a^	0.95^a^		
No. of patches (<0.10 km²)	0.46	0.47	0.46	0.54	−0.52	0.37	0.57^e^	0.56	0.63^e^	
No. of patches (<0.08 km²)	0.33	0.30	0.28	0.35	−0.27	0.23	0.40	0.33	0.42	0.89^a^

^a^*P* < 0.0001; ^b^*P* < 0.001; ^c^*P* < 0.005; ^d^*P* < 0.01; ^e^*P* < 0.05.

**Figure 2:**
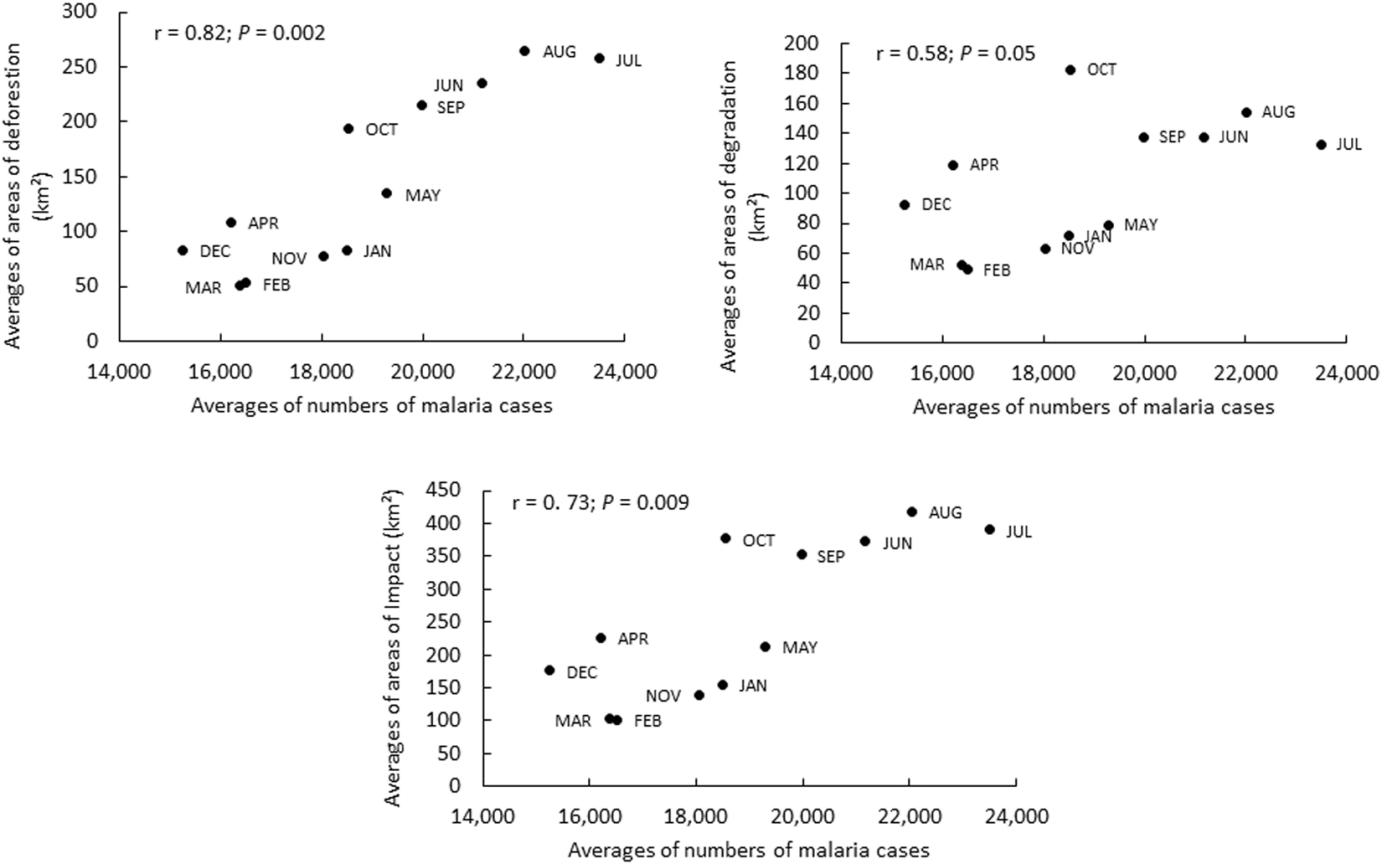
Graphs of dispersion between the number of malaria cases and the areas affected by deforestation, degradation and impacted forest with areas of less than 5 km², between 2009–2015.

In addition, between number of fragments greater than 5 km² and malaria case numbers the correlation was insignificant. Patches less than 5 km² were of particular relevance because this spatial resolution showed a significant correlation with malaria cases (0.81; *P* < 0.005) and deforestation (0.96; *P* < 0.0001), and this patch size may be biologically linked to preferred larval habitats *Ny. darlingi* (partial shade near the forest fringe)^33,44,46^. When polygons greater than 5 km² were excluded, there was a high positive correlation between deforested areas and the number of malaria cases within impacted forest (r = 0.73; *P* = 0.009). As Fig. 2 illustrates, deforestation was more strongly correlated (r = 0.82; *P* = 0.002) compared to impact and degradation was moderate (r = 0.58; *P* = 0.05).

Simple linear regression analyses showed that each km² of deforestation corresponded to an increase of 27 new malaria cases (r² = 0.78; F_1,10_ = 35.81; *P* < 0.001), whereas each km² of impacted forest corresponded to an increase of 16 new cases (r² = 0.63; F_1,10_ = 17.23; *P* = 0.001). The result of the analysis of the data on degradation was not significant (r² = 0.30; F_1,10_ = 4.37; *P* = 0.06).

Table 2 presents results of simple linear regression analyses for the variables studied. It was possible to construct three models of multiple linear regression; variables from the first model - cases and rainfall, the second model - cases and deforestation, and the third model - cases and impact. The number of patches (0 ¬ 0.10 km ²) was employed to adjust variables for the three models tested. The dependent variable was the number of malaria cases, and the independent variables were: deforestation, degradation, impact, rainfall and sizes of non-forest patches. Table 3 show annual production and value produced by forestry extraction, from 2009–2015.

**Table 2:** Models of simple and multiple linear regression for the variables about deforestation activities, landscape composition (e.g., abundance of patch units), rainfall (environmental parameter) and number of malaria cases in Brazilian Amazonian States.

Variables	β_1_ SLR coefficient (*P* value)	Model 1	β_1_ MLR adjusted (*P* value)	
			Model 2	Model 3
Deforestation (km²)	27.45 (0.00013)		23.45 (0.003)	
Degradation (km²)	31.89 (0.063)			
Impact (km²)	16.64 (0.002)			12.76 (0.02)
Accumulated rainfall (mm³)	−21.15 (0.0021)	−16.18 (0.018)		
No. of patches (>5 km²)	82.93 (0.53)			
No. of patches (<5 km²)	8.83 (0.0009)			
No. of patches (<0.5 km²)	11.73 (0.0013)			
No. of patches (<0.25 km²)	19.39 (0.004)			
No. of patches (<0.10 km²)	234.40 (0.017)	120.41 (0.14)	73.51 (0.30)	113.19 (0.18)
No. of patches (<0.08 km²)	373.90 (0.18)			

SLR: Simple Linear Regression; MLR: Multiple Linear Regression.

**Table 3:** Production and value produced by forestry extraction in the period from 2009 to 2015 in Brazilian Amazonian States and the index of value produced (USD$)/production (m^3^).

Year	Timber production (m³)	Charcoal (m^3^)	Value produced (timber)	Value produced (charcoal)	Index
2009	20,096,262	2,615,457	USD$ 764,503.88	USD$ 118,794.40	4.58
2010	18,464,248	1,964,922	USD$ 767,057.12	USD$ 100,669.87	5.16
2011	19,355,297	1,865,409	USD$ 984,770.40	USD$ 97,918.74	5.30
2012	18,642,557	1,879,235	USD$ 704,366.15	USD$ 108,184.22	5.79
2013	19,494,554	1,699,578	USD$ 865,974.44	USD$ 112,392.72	6.66
2014	18,722,351	1,949,900	USD$ 894,193.86	USD$ 132,263.72	6.83
2015	16,071,876	1,442,070	USD$ 816,336.65	USD$ 113,581.69	7.93
Total	130,847,145	13,416,570	USD$ 5,797,202.49	USD$ 783,805.37	

Note:The conversion result of the exchange value of R$ (Real) to USD$ (United States Dollar) in the period of the study (2009–2015) was as follows: R$ 1.00 corresponded to USD$ 2.18 (CI = ±0.12).

In the simple linear regression analysis, the variables that were correlated with malaria cases were: deforestation, impact, accumulated rainfall, number of patches <5 km², number of patches <0.5 km² and number of patches <0.25 km². In the multiple linear regression analysis, we constructed three models for the variable, number of malaria cases, considering the correlation between the independent variables: model 1 – with accumulated rainfall; model 2 – with deforestation and model 3 – with impact.

Statistical correlation between mean monthly malaria cases and the three levels of forest use considered in this study differed: malaria and deforestation was positive (r = 0.80; *P* = 0.002); malaria and forest impact was moderate (r = 0.56; *P* = 0.06); and malaria and forest degradation was weak (r = 0.25; *P* = 0.43). In contrast, correlation analyses using annual malaria incidence (data from Supplementary Table S1) between malaria and deforestation (km²), forest degradation (km²) and number of impacted patches were not significant (*P* = 0.30; *P* = 0.60 and *P* = 0.23 respectively).

**Figure 3:**
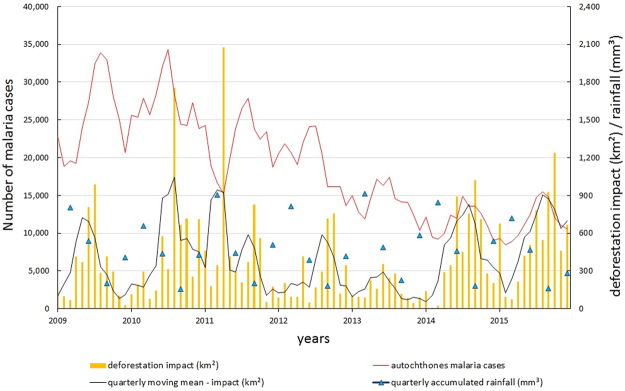
Distribution of number of cases of malaria, impacted areas of forest affected, quarterly moving mean of impacted deforestation and quarterly averages accumulated from 2009–2015 in Brazilian Amazonian States.

Spearman’s correlation tests between rainfall and deforestation (monthly means between the years 2009–2015) was negative (r = −0.88; *P* = < 0.001), where 82% of the increase in each km² of deforestation was accompanied by a reduction of each 0.95 mm³ in rainfall of the respective month (r² = 0.82; F_1,10_ = 47.22; *P* < 0.001). In contrast, the correlation with forest degradation was moderate, but not significant (r = −0.64; *P* = 0.02). When the forest impact was evaluated, the correlation was positive and significant (r² = 0.65; F_1,10_ = 18.96; *P* = 0.001). A reduction of each 0.40 mm³ in rainfall of the respective month was correlated with 65% decrease in each kilometer square of forest impact. Quarterly averages of rainfall accumulation in the Amazon region are in Fig. 3 (Supplementary Dataset S1). Maximum rainfall occurred between November and April.

**Figure 4:**
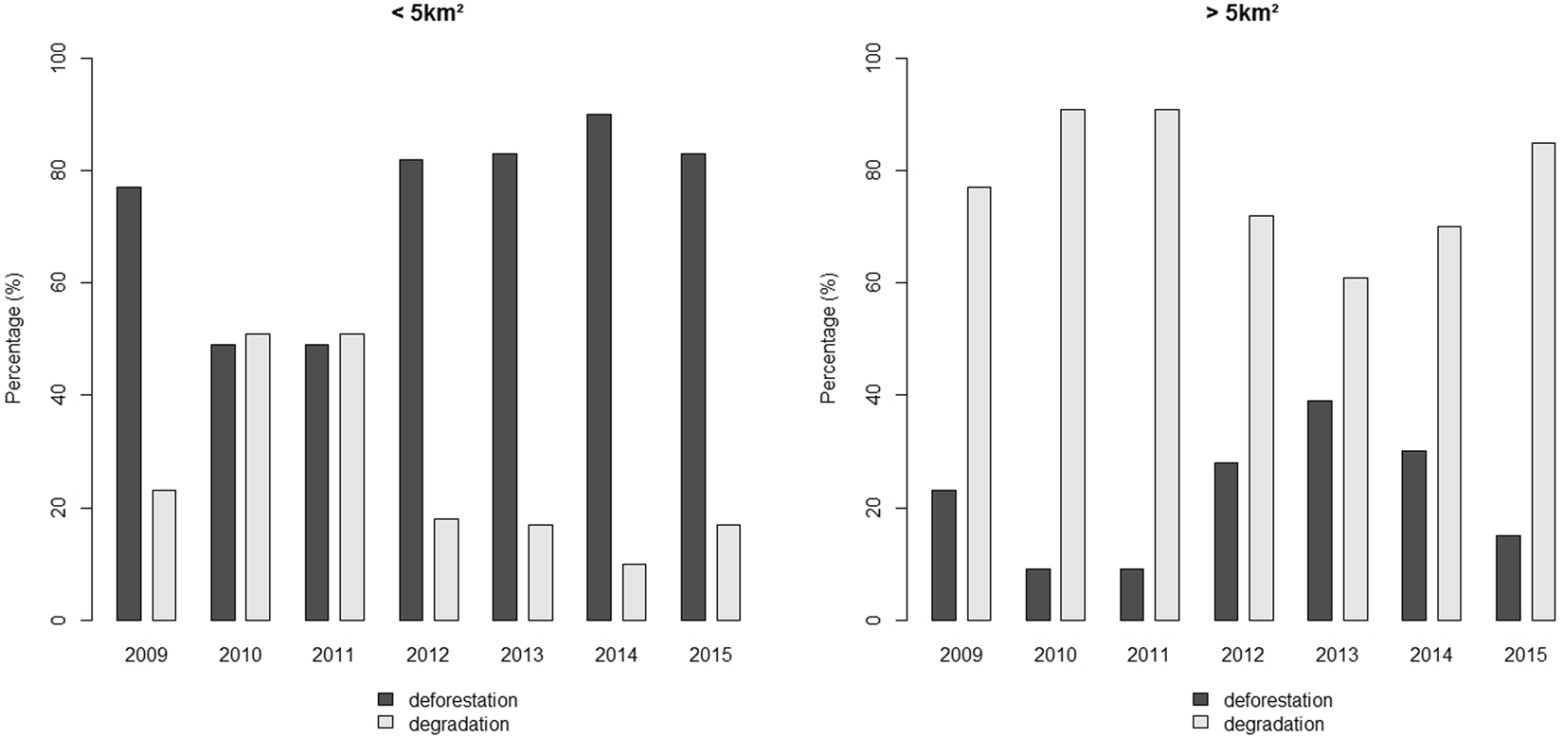
Percentage of the number of polygons of deforestation (dark grey) and degradation (light grey) with areas of less than 5 km² (left panel) and greater than 5 km² (right panel).

The Fig. 4 shows the distribution of deforested and degraded patches numbers above and less than 5 km² over the years (2009–2015). Degraded patches showed the greatest number of patches above 5 km² (right panel, Fig. 4), when compared with number of deforested patches (left panel, Fig. 4), especially in 2010 and 2011 (right panel, Fig. 4).

**Figure 5:**
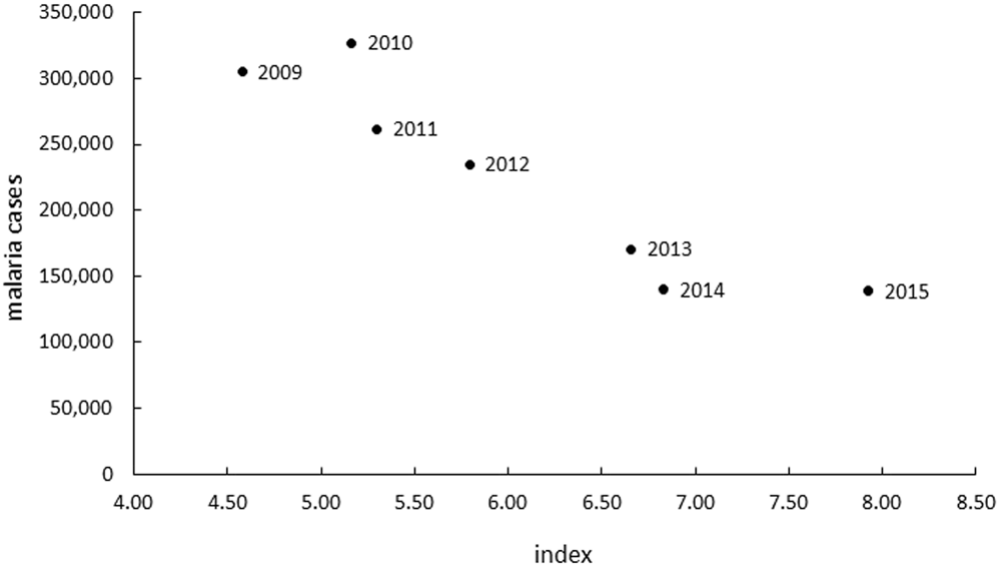
Graph of the dispersion between the total number of malaria cases and the economic index (value produced (m³)/production (m³) *100) between 2009–2015 in Brazilian Amazonian States.

Related to the exploitation of natural forestry resources, there was a highly negative correlation between the extraction forestry economic indices (value produced and total production) and the number of malaria cases (r = −0.96; P = 0.002) (Fig. 5).

The Supplementary Table S1 shows the total data per year. The greatest deforestation (3,098 km²) occurred in 2015, followed by 2014 (2,993 km²). The largest degraded area was in 2011 (4,494 km²), followed by 2010 (4,315 km²). The largest area impacted by land use transformation was observed in 2015 (6,596 km²), followed by 2011 (5,956 km²) and 2010 (5,805 km²). The greatest number of impacted polygons (patches) was observed in 2010 (7,372 patches), followed by 2009 and 2015. Monthly distribution of malaria cases, deforestation, degradation and impacted forest are found in Supplementary Figures S1 and S2.

**Figure 6:**
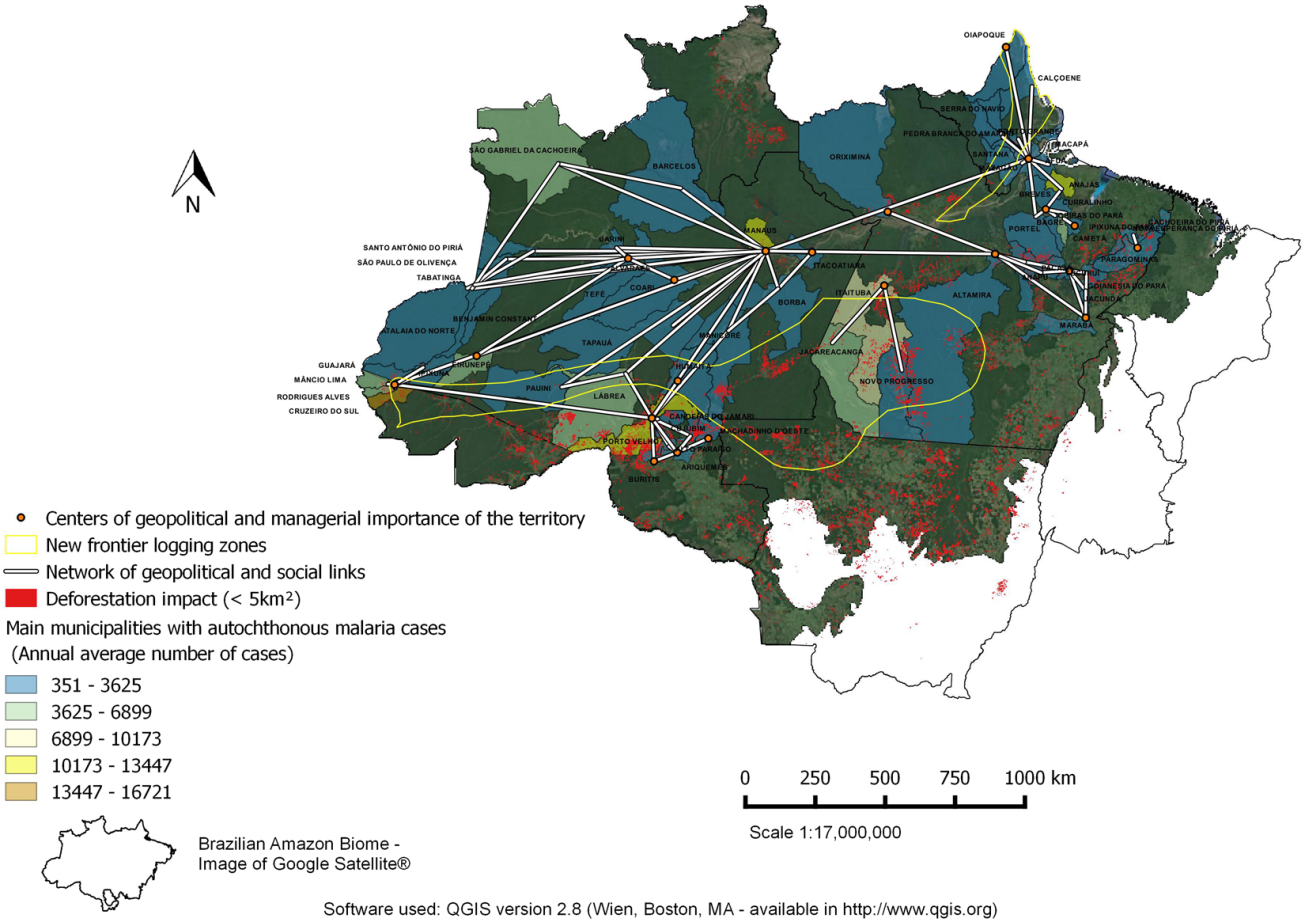
Map of the principal municipalities with autochthonous malaria cases (2009–2015), their network of geopolitical and social links, within the Brazilian Amazonian biome.

Supplementary Table S2 lists the principal municipalities with malaria cases by state. Amazonas and Pará states presented the highest annual number of malaria cases, followed by Acre, Rondônia and Amapá. Figure 6 shows the municipalities and their geopolitical and social networks. The municipalities included in the current frontier of forest exploitation (Fig. 6) accounted for 41% of malaria among all municipalities listed in Supplementary Table S2.

The annual percentage of the number of impacted forest patches of less, respectively, than 15 km², 5 km², 0.5 km² and 0.25 km² per month is in Supplementary Table S3. There was a significant decrease of more than 50% in the number of patches of less than 0.25 km² in the study period. The monthly averages of the data collected for each variable are presented in Supplementary Table S4.

## Discussion

Results of the analyses conducted for this study indicate a strong positive correlation between the number of malaria cases, deforestation and forest degradation in the Brazilian Amazon forest frontier. Furthermore, deforestation increases the risk and incidence of malaria, representing a considerable impact on malaria epidemiology. Several authors have found associations involving ecological factors and changes in land use for occupation, and the globalization of commercial and social relationships within the dynamic of infectious diseases^35,47,48^. Here we corroborated that deforestation (as defined herein) is a substantial risk factor for malaria in the Brazilian Amazon, mainly when it occurs by clearcutting areas of 5 km² or less. Therefore, decreasing the deforestation rate could be an effective measure for controlling malaria in Brazil^49^.

Taking into consideration all the results, it is plausible to assume, by analogy, a relationship of commensalism between malaria and deforestation. Malaria incidence positively increases with deforestation, benefited by a landscape that favors the presence and dispersal of Anophelinae vector species. There is a positive correlation between modifications in the natural environment of the Amazon tropical forest and the number of malaria cases. The new landscape resulting from human presence favors some species of malaria mosquitoes, whereas other species become rare or are replaced by other vector species^50^. *Nyssorhynchus darlingi*, the primary malaria vector, is positively favored by the ecology of the new human landscape, becoming abundant and dominant, especially in and around human dwelling. Furthermore, the new landscape delineated by the pattern of deforestation and soil occupation may favor dispersal of *Ny*. *darlingi* by creating forested areas interspaced by deforested areas, which are linked by forest corridors along the igarapés and shaded dirt roads such as those observed in Machadinho D’Oeste, Rondônia state, Brazil by Castro & Singer^30^.

The difference between the results of the annual and monthly correlation between malaria and deforestation analyses, confirms hypotheses by Lefèvre *et al*.^20^, Santos *et al*.^21^ and Becker *et al*.^10^, that economics and seasonal aspects are associated with the dynamics of forest frontier expansion and occurrence of malaria. In this context, the forest frontier and the malaria are elements that are linked to the spatial and temporal landscape where the drivers of the first (forest loss) are followed by an increase in the second. It was only possible to understand the dynamics involved in the behavior of the variables in our study when the data were distributed in relation to the seasonal variation of the Amazonian region. Terrazas *et al*.^5^ found a positive correlation of environmental indicators (average annual deforestation rate and percentage of areas under the influence of watercourses) and malaria incidence, strengthening the importance of implementing socioeconomic development policies articulated with actions of environmental protection and health care for the population.

It is important to highlight the actions of malaria control by the Brazilian Health Secretariat (vector control, active case detection and treatment and distribution of mosquito nets), which may have contributed to the decreased number of malaria cases in 2015. However, such a determination was beyond the scope of this study, because these activities are inconsistent and shift according to changing government policies and priorities, and available municipality budgets (for example, actions to combat dengue fever reduce malaria control activities in rural areas)^51^.

Our results verified that malaria increases along with deforestation, following the seasonality of the Amazon, driven by the rainfall regime. The latter is also an important environment component for the spread of *Ny*. *darlingi*^52^. Therefore, the annual data were not significantly correlated between malaria and the deforestation variables, because annual data hide the seasonal pattern, which is very marked in the Amazon. The driest months were associated with the largest deforested and impacted areas and the greatest numbers of malaria cases. Our findings strongly corroborate those of Kirby *et al*.^53^ who discovered that localities in the Amazon with the least rainfall and longest dry seasons were consistently more prone to deforestation, compared to those with heavier annual rainfall and shorter dry seasons.

Our major finding of a highly significant correlation between malaria incidence and patch size <5km^2^ indirectly supports the hypothesis that large deforested areas, with patches of exposed land and degraded forest larger than 5 km², are not favorable to *Ny*. *darlingi*, a vector that uses the forest edge for maintenance of its immature forms^26,54^. Barros *et al*.^33^ verified that malaria was correlated with shorter distances to potential transmission hotpots and people living within 400 m of such hotspots had a 2.60 higher risk of malaria. One apparent discrepancy in our findings was that April 2011 registered a large impacted area (2,074 km²), though the number of malaria cases that month was smaller (15,267 cases) than that of the previous month (March/2011: 16,615 cases). The most likely reason for this apparent discrepancy is that a greater number of patches >5 km² accounted for the smaller number of cases in April, i.e., only 8% of the total number of polygons accounted for 62.8% of the total area impacted. This observation reinforces the role of small patches in malaria epidemiology.

Decisions and delimitation of areas that will be used to develop and start a new rural settlements are undertaken, as a rule, in the absence of specific projects capable of generating income, improving socioeconomic growth and wellbeing, or promoting environmental sustainability of the rural communities involved^55^. It appears that localization of malaria cases and areas of deforestation are highly sensitive to Indirect Land Use Change (ILUC), a phenomenon that occurs when agricultural activities are transferred from one region and reconstituted in another^56^. Arima *et al*.^57^ observed that displacement of cattle breeding, due to agricultural expansion, lies behind changes in land use in municipalities hundreds of kilometers away. This is driven by land speculation, a search for basic supplements such as wood for fences or house construction, migration and a demand for labor. Thus, both deforestation and malaria may be being determined, in part, by distant events.

Our analyses of the economic data emphasize the forces that have expanded the scope of deforestation in the search for commercially valuable forest resources. The abundance of a particular resource leads to its rapid exploitation, which, in turn, leads to its long-term decline either by virtue of its scarcity or because the offer exceeds the demand, thus lowering its domestic and foreign market value^15^. With the increase in demand, the mechanism of the market will lead to a rise in the product price, attracting a larger number of producers and encouraging additional deforestation.

A closer look at the networks of geopolitical and social links among the municipalities (Fig. 6), strengthens the premise that malaria in the Amazon may be subject to deforestation activities hundreds of kilometers away. Schneider & Peres^58^ demonstrate that rural Amazonian settlements have increased deforestation and among the principal contributory activities they highlight timber, firewood and charcoal production. The authors observed that timber and firewood production begins to increase well before the formal launching of the settlements, timber exploitation increases for 5 years after the official launch, then falls abruptly after 9 years, probably due to a lack of wood of adequate market value. During this process, charcoal production grows rapidly from initiation until a settlement completes an estimated 6 years of activity, then abruptly falls, characterizing a final phase, lasting until the opening of new fronts of deforestation.

Results of our economic index analysis demonstrate that the greater the production in terms of value produced, the lower the index, and the greater the correlation with the number of cases of malaria. Hahn and collegues^49^ found that over half of the municipalities with forest exploitation for timber production were also affected by an additional 7% selective logging that was causing deforestation in areas of preserved forests. The selective forest wood resources exploitation is associated with an increase in malaria^49^ and further supports the frontier malaria concept.

Frontier malaria has been defined by Sawyer^1,6,59^, Singer & Castro^22^, Castro *et al*.^23^ and Castro & Singer^30^ as a process associated with settlements, characterized by environmental disturbance, increased vector abundance, primary forest reduction for agriculture, and the establishment of precarious communities. This process causes obvious changes in land use for human occupation. The settlers’ demands of land for agriculture and timber for firewood, fencing and house construction lead inevitably to deforestation.

There are interesting parallels between the occupation of the Brazilian Atlantic Forest in the 1920’s^60,61^ for coffee plantation and firewood exploitation, and the present Amazon Basin occupation for beef and soybean production^62^. If in the early 20th century malaria incidence had been more than 80% in the Atlantic Forest^63^, today, with only 12.5% of the original forest cover remaining (https://www.sosma.org.br), malaria migrated together with deforestation to the Amazon, where more than 99% of malaria cases currently occur^63^ and represent one of the biggest deforestation fronts in the world (http://wwf.panda.org). With an average predominance of 60% of patches less than 0.5 km², our study showed that deforestation is a major pathway for malaria cases in the Amazon.

Many factors contribute to the challenge of malaria control, for instance, there are other vector species competent to transmit *Plasmodium* and adapted to deposit eggs in open areas with partial shade, such as *Ny*. *marajoara* (Galvão & Damasceno)^50,64,65^, and *Ny. deaneorum* (Rosa-Freitas)^66^. These species have distinctive ecologies compared with *Ny*. *darlingi* and require alternative strategies for control. Human migration into areas of malaria risk to start a new rural settlements is also an important determinant of malaria incidence^67^. In Brazil, human migration into endemic transmission areas is promoted by the Brazilian’s National Institute of Colonization and Agrarian Reform (Instituto Nacional de Colonização e Reforma Agrária - Incra), responsible for a national landholder program whose main pillar is land distribution in rural areas to decrease poverty and decrease unemployment rate in Brazil. In this context, frontier malaria operates at three spatial scales. At the lowest, or micro scale, there is increased anopheline vector abundance resulting from a range of disturbances in the ecosystem, and increased human exposure as a consequence of inadequate housing^68^. At the community scale, weak institutions, marginalized settlers, high rates of in and out-migration, and human mobility together ensure the proliferation of *Plasmodium* parasites^24,30^. The national scale is characterized by an unplanned development program of land occupation based on distribution of small land properties^30^.

Our findings also present the opportunity to promote the maintenance of ecosystem services rendered by the Amazonian forest on all geographical levels, here considered to be an important aspect of the control of malaria in Brazil. Just as the identification of hotspots of threatened species is an essential approach for setting conservation priorities, production and consumption of global goods and services can be connected through commercial geopolitical links^69^. Austin *et al*.^70^ found a positive associations between deforestation rates and malaria prevalence across 67 nations and suggest that anthropogenic drivers of environmental degradation (rural population growth and specialization in agriculture) are an important factor to consider in explaining cross-national variation in malaria rates. Therefore, locating malaria driven by the global consumption of goods and services can help to connect epidemiological surveillance, supplies and demands, companies and governments in order to better target malaria control actions in futures research.

## Conclusions

Deforestation is an major risk factor for malaria in the Brazilian Amazonia, mainly when it occurs in areas of less than 5 km². This activity directly or indirectly results in a low Human Development Index (HDI) and environmental conditions favorable to vector proliferation. Despite recent advances in vaccine development^71^ and the effective treatment of malaria with new drugs and techniques^72^, vector interventions, adequate planning of land use and occupation, and promotion of the minimum conditions necessary for the generation of income for the subsistence and financial autonomy of humans living in the Amazon, are sadly lacking and need to become a priority to prevent forest ecosystem failure and further public health discrepancies.

Supplementary information is available for this paper. The taxonomic nomenclature of Anophelinae followed that proposed by Foster *et al*.^73^ Accordingly, except in references *Anopheles* (*Nyssorhynchus*) *darlingi* Root is herein referred as *Nyssorhynchus darlingi* (Root).

## Electronic supplementary material

Supplementary Tables and Figures

Supplementary Tables

Supplementary Dataset 1

## References

[1] Sawyer DR (1986). Malaria on the Amazon frontier: economic and social aspects of transmission and control. Southeast Asian J. Trop. Med. Public Health.

[2] Valle D, Clark J (2013). Conservation efforts may increase malaria burden in the Brazilian Amazon. PLoS One.

[3] Olson SH, Gangnon R, Silveira GA, Patz JA (2010). Deforestation and malaria in Mancio Lima county, Brazil. Emerging Infect. Dis..

[4] Bauch SC, Birkenbach AM, Pattanayak SK, Sills EO (2015). Public health impacts of ecosystem change in the Brazilian Amazon. Proc. Natl. Acad. Sci. USA.

[5] Terrazas WCM (2015). Deforestation, drainage network, indigenous status, and geographical differences of malaria in the State of Amazonas. Malaria J..

[6] Sawyer, D. R. Frontier malaria in the Amazon region of Brazil: types of malaria situations and some implications for control. Brasília: PHO/WHO/TDR (1988).

[7] Lima JMT, Vittor A, Rifai S, Valle D (2017). Does deforestation promote or inhibit malaria transmission in the Amazon? A systematic literature review and critical appraisal of current evidence. Phil. Trans. R. Soc. B..

[8] Fernandes, V. B. & Reydon, B. P. Sobre o conceito de fronteira. In: “Desenvolvimento sem desmatamento”. Policy in focus – Centro Internacional de Políticas para o Crescimento Inclusivo (IPC-IG), Programa das Nações Unidas para o Desenvolvimento. Agosto, No. 29 (2014).

[9] UNCTAD – United Nations Conference on Trade and Development. 2015. State of Commodity Dependence. Special Unit on Commodities. United Nations, New York and Geneva (2014).

[10] Becker, B. K., Geopolítica da Amazônia. Conferência do Instituto de Estudos Avançados da USP. Abril, **19**(53) (2005).

[11] Sasaki N, Putz FE (2009). Critical need for new definitions of “forest” and “forest degradation” in global climate change agreements. Conserv. Lett..

[12] Turner M, Donato D, Romme W (2013). Consequences of spatial heterogeneity for ecosystem services in changing forest landscapes: priorities for future research. Landsc. Ecol..

[13] Mitchell M (2015). Reframing landscape fragmentation’s effects on ecosystem services. Trends Ecol. Evol..

[14] CPT – Comissão Pastoral da Terra. Por debaixo da floresta: Amazônia paraense saqueada com trabalho escravo. Coordenação: Centro de Defesa da Vida e dos Direitos Humanos Carmen Bascarán. Projeto Raice – Rede de Ação Integrada para Combater a Escravidão, Fase 1: Diagnóstico. Araguaína, Tocantins, 90 p. (2016).

[15] Sant’Anna AA, Young CEF (2010). Property Rights. Deforestation and Rural Conflicts in the Amazon. Economia Aplicada v. 14, n..

[16] L’Roe J, Rausch L, Munger J, Gibbs H (2016). Mapping properties to monitor forests: Landholder response to a large environmental registration program in the Brazilian Amazon. Land Use Policy.

[17] Young CEF (2006). Desmatamento e desemprego rural na Mata Atlântica. Floresta e Ambiente v..

[18] Sant’Anna AA (2017). Land inequality and deforestation in the Brazilian Amazon. Environ. Dev. Econ..

[19] IBGE – Instituto Brasileiro de Geografia e Estatística. Produção da Extração Vegetal e da Silvicultura. Rio de Janeiro. v. 30, p. 1–48 (2015).

[20] Lefèvre T (2009). Beyond nature and nurture: phenotypic plasticity in blood-feeding behavior of Anopheles gambiae s.s. when humans are not readily accessible. *Am*. *J*. Trop. M..

[21] Santos LM, Gama RA, Eiras AE, Fonseca CG (2010). Genetic differences based on AFLP markers in the mosquito species *Anopheles darlingi* collected in versus near houses in the region of Porto Velho, RO, Brazil. Genet. Mol. Res..

[22] Singer BH, De Castro MC (2001). Agricultural colonization and malaria on the Amazon frontier. *Ann*. *N*. Y. Acad. Sci. Dec..

[23] Castro MC, Monte-Mor RL, Sawyer DO, Singer BH (2006). Malaria risk on the Amazon Frontier. Proc. Natl. Acad. Sci. USA.

[24] Carrasco-Escobar G (2017). Micro-epidemiology and spatial heterogeneity of *P*. *vivax* parasitaemia in riverine communities of the Peruvian Amazon: A multilevel analysis. Sci. Rep..

[25] Galardo AKR (2009). Seasonal abundance of anopheline mosquitoes and their association with rainfall and malaria along the Matapi River, Amapi, Brazil. Med. Vet. Entomol..

[26] Barros FSM, Arruda ME, Gurgel HC, Honorio NA (2011). Spatial clustering and longitudinal variation of Anopheles darlingi (Diptera: Culicidae) larvae in a river of the Amazon: the importance of the forest fringe and of obstructions to flow in frontier malaria. B. Entomol. Res..

[27] Valle D, Lima JMT (2014). Large-scale drivers of malaria and priority areas for prevention and control in the Brazilian Amazon region using a novel multi-pathogen geospatial model. Malar. J..

[28] Walsh JF, Molyneux DH, Birley MH (1993). Deforestation: effects on vector-borne disease. Parasitol..

[29] Lana RM (2017). Socioeconomic and demographic characterization of an endemic malaria region in Brazil by multiple correspondence analysis. Malar. J..

[30] Castro, M. C. & Singer B. H. Malária in the Brazilian Amazon. In: Water and Sanitation-Related Diseases and the Environment: Challenges, Interventions and Preventive Measures 19 p. (2011).

[31] Angelo JR (2017). The role of spatial mobility in malaria transmission in the Brazilian Amazon: The case of Porto Velho municipality, Rondônia, Brazil (2010–2012). PloS One.

[32] Costa S, Brondizio E (2009). Dependência Inter-urbana entre as Cidades Amazônicas: Crescimento Urbano, Deficiências em Infra-estrutura e Redes Sociais. REDES.

[33] Barros FS, Honório NA (2015). Deforestation and malaria on the amazon frontier: larval clustering of Anopheles darlingi (Diptera: Culicidae) determines focal distribution of malaria. Am. J. Trop. Med. Hyg..

[34] Hiwat H, Bretas G (2011). Ecology of Anopheles darlingi Root with respect to vector importance: a review. Parasit. Vectors.

[35] Yasuoka J, Levins R (2007). Impact of deforestation and agricultural development on anopheline ecology and malaria epidemiology. Am. J. Trop. Med. Hyg..

[36] Gil LHS, Rodrigues MDS, Katsuragawa TH (2015). Seasonal distribution of malaria vectors (Diptera: Culicidae) in rural localities of Porto Velho, Rondônia, Brazilian Amazon. *Revi*. *I*. Med. Trop. São Paulo.

[37] Kweka EJ, Kimaro EE, Munga S (2016). Effect of Deforestation and Land Use Changes on Mosquito Productivity and Development in Western Kenya Highlands: Implication for Malaria Risk. Front Public Health.

[38] Vittor AY (2006). The effect of deforestation on the human-biting rate of *Anopheles darlingi*, the primary vector of falciparum malaria in the Peruvian Amazon. Am. J. Trop. Med. Hyg..

[39] Asner GP (2006). Condition and fate of logged forests in the Brazilian Amazon. Proc. Natl. Acad. Sci. USA.

[40] Da Silva C. A. & De Souza, C. M. Jr. Comparação entre imagens Landsat ETM+e MODIS/Terra para detecção de incrementos de desmatamento na região do Baixo Acre. *Revista Brasileira de Cartografia* **2**(57) (2005).

[41] De Souza Jr, C. M., Hayashi, S. & Veríssimo, A. Near real-time deforestation detection for enforcement of forest reserves in Mato Grosso. In Proceedings of Land Governance in Support of the Millennium Development Goals: Responding to New Challenges, World Bank Conference, Washington, DC (2009).

[42] Zhu S, Zhang H, Liu R, Cao Y, Zhang G (2014). Comparison of Sampling Designs for Estimating Deforestation from Landsat TM and MODIS Imagery: A Case Study in Mato Grosso, Brazil. Sci. World J..

[43] Broich M, Stehman S, Hansen M, Potapov P, Shimabukuro Y (2009). A comparison of sampling designs for estimating deforestation from Landsat imagery: A case study of the Brazilian Legal Amazon. Remote Sen. Environ..

[44] Adde A (2016). Dynamical Mapping of *Anopheles darlingi* Densities in a Residual Malaria Transmission Area of French Guiana by Using Remote Sensing and Meteorological Data. PLoS One.

[45] Barona E, Ramankutty N, Hyman G, Coomes OT (2010). The role of pasture and soybean in deforestation of the Brazilian Amazon. Environ. Res. Lett..

[46] Ladeia-Andrade S (2007). Naturally acquired antibodies to merozoite surface protein (MSP)-1(19) and cumulative exposure to *Plasmodium falciparum* and *Plasmodium vivax* in remote populations of the Amazon Basin of Brazil. *Mem*. *I*. Oswaldo Cruz.

[47] Kilpatrick AM, Randolph SE (2012). Drivers, dynamics, and control of emerging vector-borne zoonotic diseases. Lancet.

[48] Prist PR (2016). Landscape, Environmental and Social Predictors of Hantavirus Risk in São Paulo, Brazil. PloS One.

[49] Hahn, M. B., Gangnon, R. E., Barcellos, C., Asner, G. P. & Patz, J. A. Influence of deforestation, logging, and fire on malaria in the Brazilian Amazon. *PLoS One* **9**(1) (2014). 10.1371/journal.pone.0085725 PMC3880339 24404206

[50] Conn JE (2002). Emergence of a new neotropical malaria vector facilitated by human migration and changes in land use. Am. J. Trop. Med. Hyg..

[51] Suárez-Mutis MC, Coura JR (2007). Mudanças no padrão epidemiológico da malária em área rural do médio Rio Negro, Amazônia brasileira: análise retrospectiva. Cad. Saude Publica.

[52] Magris M, Rubio-Palis Y, Menares C, Villegas L (2007). Vector bionomics and malaria transmission in the Upper Orinoco River, Southern Venezuela. *Mem*. *I*. Oswaldo Cruz.

[53] Kirby KR (2006). The future of deforestation in the Brazilian Amazon. Futures.

[54] Charlwood JD (1996). Biological variation in Anopheles darlingi Root. *Mem*. *I*. Oswaldo Cruz.

[55] Soares, J. L. N. Estudo acerca da capacidade de geração de renda do projeto de assentamento Serragem/Santana. Estudo apresentado à Divisão de Obtenção de Terras e Implantação de Projetos de Assentamento do INCRA SR (01), atendendo Resolução MDA/INCRA No. 5 de 29 de março de 2012 (2013).

[56] Lapola DM (2010). Indirect land-use changes can overcome carbon savings from biofuels in Brazil. Proc. Natl. Acad. Sci. USA.

[57] Arima EY, Richards P, Walker R, Caldas MM (2011). Statistical confirmation of indirect land use change in the Brazilian Amazon. Environ. Res. Lett..

[58] Schneider M, Peres CA (2015). Environmental costs of government-sponsored agrarian settlements in Brazilian Amazonia. PloS One.

[59] Sawyer, D. R., & Sawyer, D. O. The malaria transition and the role of social science research. Advancing the health in developing countries: the role of social research. Westport: Auburn House, **105**, 122 (1992).

[60] Kronka, F. J. N. *et al*. Inventário florestal da vegetação nativa do Estado de São Paulo. Secretaria do Meio Ambiente, Instituto Florestal. 200 p. (2005).

[61] Dean, W. A ferro e fogo: a história e a devastação da Mata Atlântica brasileira. 1ª Ed. São Paulo: Cia. das Letras, 484 p. (2004).

[62] Richards PD, Walker RT, Arima EY (2014). Spatially complex land change: The Indirect effect of Brazil’s agricultural sector on land use in Amazonia. Glob. Environ. Change.

[63] De Pina-Costa A (2014). Malaria in Brazil: what happens outside the Amazonian endemic region. *Mem*. *I*. Oswaldo Cruz.

[64] Galardo AKR (2007). Malaria vector incrimination in three rural riverine villages in the Brazilian Amazon. Am. J. Trop. Med. Hyg..

[65] Moreno, M. *et al*. Intensive trapping of blood-fed *Anopheles darlingi* in Amazonian Peru reveals unexpectedly high proportions of avian blood-meals. *PLoS Neglect*. *Trop*. *D*. **11**(2) (2017). 10.1371/journal.pntd.0005337 PMC5322880 28231248

[66] Laporta GZ (2015). Malaria vectors in South America: current and future scenarios. Parasites & Vectors.

[67] Marques AC (1987). Human migration and the spread of malaria in Brazil. Parasitol. Today.

[68] Reis IC (2015). Epidemic and endemic malaria transmission related to fish farming ponds in the Amazon frontier. PLoS One.

[69] Moran D, Kanemoto K (2017). Identifying species threat hotspots from global supply chains. Nature Ecology & Evolution.

[70] Austin K, Bellinger M, Rana P (2017). Anthropogenic forest loss and malaria prevalence: a comparative examination of the causes and disease consequences of deforestation in developing nations. Aims Environ. Sci..

[71] Li X (2016). Human CD8+ T cells mediate protective immunity induced by a human malaria vaccine in human immune system mice. Vaccine.

[72] Organization WH. Immunization, Vaccines andBiologicals [Available from: http://www.who.int/vaccine_research/links/Rainbow/en/index.html (2017).

[73] Foster PG (2017). Phylogeny of Anophelinae using mitochondrial protein coding genes. R. Soc. Open Sci..

