# Supplementary material for: Abundance of impacted forest patches less than 5 km2 is a key driver of the incidence of malaria in Amazonian Brazil
Source: Sci Rep. 2018 May 4;8:7077. doi: 10.1038/s41598-018-25344-5 (PMC5935754; doi:10.1038/s41598-018-25344-5)
Supplement: Supplementary file 1 — Supplementary Tables and Figures [file 41598_2018_25344_MOESM1_ESM.pdf]

Abundance of impacted forest patches less than 5 km^2 is a key driver of the incidence of malaria in Amazonian Brazil

Leonardo Suveges Moreira Chaves · Jan E Conn · Rossana Verónica Mendoza López · Maria Anice Mureb Sallum

Supplementary Information

Definitions used in text
• **Frontier malaria** - malaria associated with anthropogenic activities that include deforestation, a process that involves ecological phenomena, acting at multiple scales, together with human behavioral and economic conditions.<br>• **Deforestation** - a human-induced process whereby the rate of clearcutting forest over time, where the forest will regenerate itself, is associated with the changes in land use and land occupation by humans often follows.<br>• **Degraded forest** - a natural forest that has been altered at any level by human activities; change that negatively affects the structure or function of forest ecosystem components, the degree of human disturbance and openness of the forest canopy for logging.<br>• **Impacted forest** - the sum of the deforested and forest degraded areas detected by the Imazon© system.<br>• **Selective logging** - logging exploitation of a few commercial trees species, leaving most of the forest in place; however, forests become very degraded during these operations.<br>• **Ecosystem services** - the benefits people obtain from ecosystems, currently categorized into four types: provisioning, regulating, cultural, and supporting services.

Supplementary Table S1. Total number of malaria cases; total deforested, degraded and impacted areas; and total number of patches modified in Brazilian Amazonian states between 2009-2015.

**Year**	**No. of malaria cases**	**Deforestation (km²)**	**Degradation (km²)**	**Impacted forest (km²)**	**Number of impacted patches**
2009	304,823	1,893	2,115	4,008	6,577
2010	327,142	1,490	4,315	5,805	7,372
2011	261,667	1,462	4,494	5,956	6,101
2012	234,967	1,768	1,557	3,325	2,767
2013	170,588	1,144	738	1,882	2,023
2014	139,935	2,993	2,132	5,125	5,614
2015	138,697	3,098	3,498	6,596	6,235
Total	1,577,819	13,847	18,850	32,698	36,689

Supplementary Table S3. Annual percentage corresponding to the number of impacted patches than 15 km², 5 km², 0.5 km² and 0.25 km² for each month.

**Months**	**Impacted patches**	**2009**	**2010**	**2011**	**2012**	**2013**	**2014**	**2015**
**January**	< 15 km²	99%	100%	99%	100%	99%	99%	99%
	< 5 km²	96%	100%	96%	98%	95%	98%	95%
	< 0.5 km²	45%	86%	67%	70%	64%	70%	51%
	< 0.25 km²	23%	64%	41%	37%	39%	36%	22%

**February**	< 15 km²	100%	100%	100%	100%	100%	100%	100%
	< 5 km²	99%	98%	100%	100%	87%	100%	96%
	< 0.5 km²	62%	65%	73%	51%	22%	95%	60%
	< 0.25 km²	34%	36%	41%	23%	9%	60%	32%
**March**	< 15 km²	100%	99%	95%	98%	97%	95%	99%
	< 5 km²	100%	99%	95%	98%	97%	95%	99%
	< 0.5 km²	85%	66%	64%	52%	58%	64%	49%
	< 0.25 km²	60%	32%	33%	24%	30%	14%	20%
**April**	< 15 km²	100%	100%	97%	98%	100%	97%	99%
	< 5 km²	97%	100%	92%	93%	97%	87%	99%
	< 0.5 km²	58%	72%	52%	36%	68%	18%	36%
	< 0.25 km²	37%	30%	28%	9%	39%	5%	12%
**May**	< 15 km²	100%	100%	100%	96%	100%	100%	100%
	< 5 km²	98%	100%	98%	85%	98%	95%	99%
	< 0.5 km²	55%	64%	68%	27%	47%	50%	44%
	< 0.25 km²	33%	22%	36%	9%	20%	20%	14%
**June**	< 15 km²	99%	100%	100%	100%	100%	100%	100%
	< 5 km²	95%	98%	99%	100%	96%	98%	99%
	< 0.5 km²	45%	60%	76%	44%	43%	52%	54%
	< 0.25 km²	24%	23%	39%	10%	16%	20%	17%
**July**	< 15 km²	100%	100%	100%	100%	100%	100%	100%
	< 5 km²	99%	99%	100%	98%	97%	99%	99%
	< 0.5 km²	68%	82%	79%	64%	58%	73%	68%
	< 0.25 km²	43%	49%	45%	42%	26%	36%	29%
**August**	< 15 km²	100%	99%	100%	100%	100%	99%	100%
	< 5 km²	100%	97%	100%	98%	95%	98%	99%
	< 0.5 km²	89%	68%	85%	37%	61%	62%	70%
	< 0.25 km²	57%	41%	50%	12%	25%	28%	41%

**September**	< 15 km^2	100%	99%	99%	99%	100%	99%	98%
	< 5 km^2	99%	96%	95%	95%	100%	97%	94%
	< 0.5 km^2	85%	72%	65%	56%	57%	63%	58%
	< 0.25 km^2	57%	44%	34%	33%	15%	27%	28%
**October**	< 15 km^2	100%	100%	98%	99%	100%	99%	99%
	< 5 km^2	100%	99%	95%	97%	97%	96%	94%
	< 0.5 km^2	82%	66%	63%	44%	72%	58%	61%
	< 0.25 km^2	48%	40%	35%	16%	26%	23%	37%
**November**	< 15 km^2	100%	100%	100%	100%	100%	100%	99%
	< 5 km^2	100%	97%	96%	99%	100%	99%	97%
	< 0.5 km^2	84%	60%	53%	65%	75%	77%	71%
	< 0.25 km^2	47%	35%	23%	36%	48%	41%	46%
**December**	< 15 km^2	100%	100%	99%	98%	100%	99%	97%
	< 5 km^2	100%	98%	96%	86%	98%	95%	93%
	< 0.5 km^2	77%	67%	48%	37%	80%	64%	39%
	< 0.25 km^2	59%	38%	19%	20%	49%	37%	9%

Supplementary Table S4. Monthly averages of variables database analyzed between 2009 - 2015 in Brazilian Amazonian States.

Averages of variables database between 2009 and 2015

**Months**	**No. of malaria cases**	**Deforestation (km²)**	**Degradation (km²)**	**Impact (km²)**	**Accumulated rainfall (mm³)**	**No. of patches (5.00 ¬ 180.00 km²)**	**No. of patches (0.05 ¬ 5.00 km²)**	**No. of patches (0.05 ¬ 0.50 km²)**	**No. of patches (0.05 ¬ 0.25 km²)**	**No. of patches (0.05 ¬ 0.10 km²)**	**No. of patches (0.05 ¬ 0.08 km²)**
JAN	18,497.7	83.3	71.4	154.6	297.8	8.3	241.0	161.4	93.3	13.6	8.6
FEB	16,508.0	53.2	48.8	102	314.8	2.9	170.9	108.4	58.1	3.1	2.4

MAR	16,368.9	50.2	52	102.3	314.5	3.7	170.6	115.4	62.7	5.7	2.1
APR	16,220.0	108.1	118.8	227	243.2	16.9	292.7	166.1	89.7	9.6	5.9
MAY	19,286.9	134.7	78.6	213.3	183.7	8.6	304.4	172.1	80.0	5.4	2.3
JUN	21,165.0	234.9	137.5	372.4	100.2	11.6	524.6	293.6	121.3	5.0	1.4
JUL	23,507.9	257.5	132.4	390.0	79.3	8.0	734.4	526.0	287.3	27.1	9.7
AUG	22,030.7	265.0	153.7	418.7	65.9	13.7	861.7	627.3	359.6	13.6	3.7
SEP	19,975.4	215.0	137.7	352.7	69.5	22.1	646.1	453.9	249.0	20.7	7.0

OCT	18,537.4	193.8	182.8	376.6	131.7	19.3	618.4	407.6	220.3	8.4	3.7
NOV	18,045.9	76.8	62.8	139.6	170.3	4.6	276.0	202.6	117.4	6.1	2.9
DEZ	15,259.0	83.5	92.6	176.1	218.2	10.4	270.4	171.1	93.4	5.3	2.1

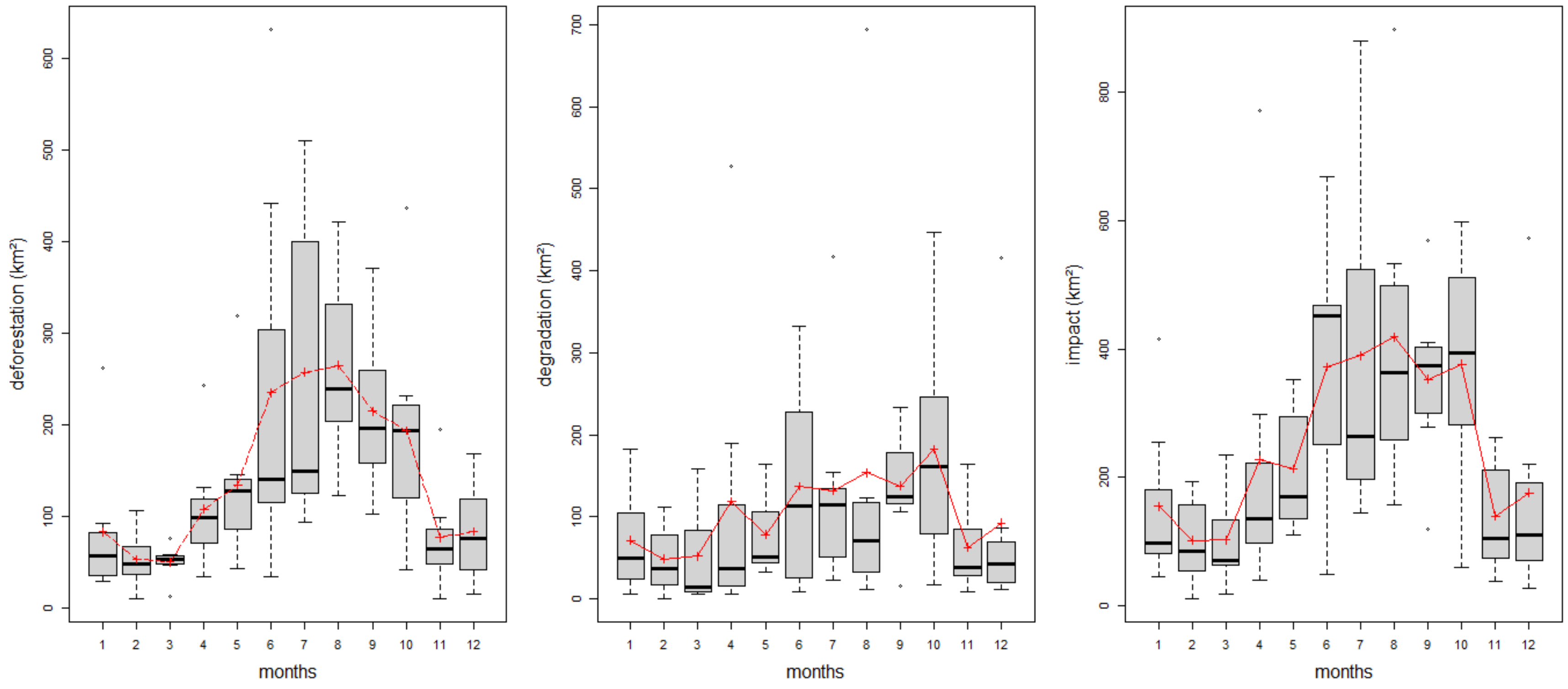

Supplementary Figure S1. Boxplot graphs of the data on deforestation, degradation and forestry impact between 2009 - 2015.

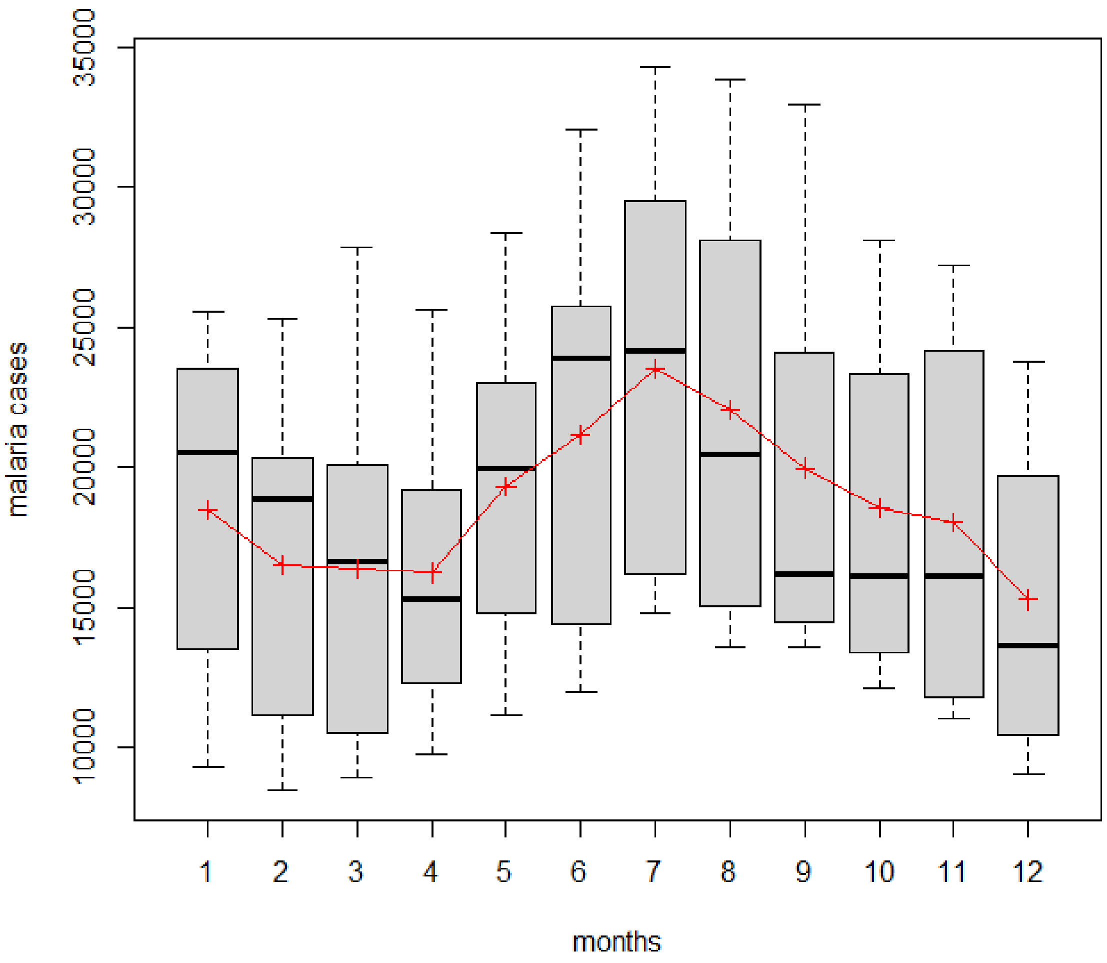

Supplementary Figure S2. Boxplot graphs of the monthly data on number of malaria cases between 2009 and 2015.
