# Supplementary material for: Abundance of impacted forest patches less than 5 km2 is a key driver of the incidence of malaria in Amazonian Brazil
Source: Sci Rep. 2018 May 4;8:7077. doi: 10.1038/s41598-018-25344-5 (PMC5935754; doi:10.1038/s41598-018-25344-5)
Supplement: Supplementary file 2 — Supplementary Tables [file 41598_2018_25344_MOESM2_ESM.pdf]

Abundance of impacted forest patches less than 5 km² is a key driver of the incidence of malaria in Amazonian Brazil

Leonardo Suveges Moreira Chaves · Jan E Conn · Rossana Verónica Mendoza López · Maria Anice Mureb Sallum

Supplementary Information

Supplementary Table S2. Principal municipalities with malaria cases in Brazilian Amazonia: total number of cases, annual averages, their share in the number of cases of their respective State, managerial centers of the territory and network of geopolitical and social influence.

States / Municipalities	Malaria cases							Annual average (Malaria cases)	Share of the Municipalities in the States (%)							Managerial centers of the territory [1]	Municípalities of geopolítical and social influence
	2009	2010	2011	2012	2013	2014	2015		2009	2010	2011	2012	2013	2014	2015		
ACRE	25.762	35.783	21.958	27.330	33.658	30.757	26.595	28.835									
Cruzeiro do Sul	15.161	21.790	12.689	15.993	19.602	17.186	14.629	16.721	58.85	60.89	57.79	58.52	58.24	55.88	55.01	Sub-regional center B (3b)	Manaus, Porto Velho, Mâncio Lima, Rodrigues Alves
Mâncio Lima	5.010	5.722	4.850	5.271	7.393	6.285	5.570	5.729	19.45	15.99	22.09	19.29	21.97	20.43	20.94	Local Center (5)	Cruzeiro do Sul

States / Municipalities	Malaria cases 2009	2010	2011	2012	2013	2014	2015	Annual average (Malaria cases)	Share of the Municipalities in the States (%) 2009	2010	2011	2012	2013	2014	2015	Managerial centers of the territory [1]	Munícipalities of geopolítical and social influence
Rodrigues Alves	2.517	4.352	3.367	3.384	3.513	4.768	4.374	3.754	9.77	12.16	15.33	12.38	10.44	15.50	16.45	Local Center (5)	Cruzeiro do Sul
Total									**88.07**	**89.05**	**95.21**	**90.19**	**90.64**	**91.81**	**92.40**		
AMAPÁ	12.448	12.260	16.926	12.893	13.326	12.709	12.968	13.361									
Macapá	2.316	1.823	2.886	2.653	3.500	1.725	2.316	2.460	18.61	14.87	17.05	20.58	26.26	13.57	17.86	Regional Capital C (2c)	Manaus, Oiapoque, Santana, Calçoene, Porto Grande, Mazagão, Serra do Navio, Portel, Anajás
Oiapoque	3.387	3.963	4.240	2.363	1.242	654	1.059	2.415	27.21	32.32	25.05	18.33	9.32	5.15	8.17	Local Centrer (5)	Macapá
Santana	1.772	1.422	2.247	1.111	1.436	2.469	1.713	1.739	14.24	11.60	13.28	8.62	10.78	19.43	13.21	Local Center (5)	Macapá

States / Municipalities	Malaria cases							Annual average (Malaria cases)	Share of the Municipalities in the States (%)							Managerial centers of the territory [1]	Munícipalities of geopolítical and social influence
	2009	2010	2011	2012	2013	2014	2015		2009	2010	2011	2012	2013	2014	2015		
Porto Grande	1.909	1.979	2.194	1.423	575	1.178	826	1.441	15.34	16.14	12.96	11.04	4.31	9.27	6.37	Local Center (5)	Macapá
Mazagão	488	656	1.450	1.806	2.210	1.599	1.335	1.363	3.92	5.35	8.57	14.01	16.58	12.58	10.29	Local Center (5)	Macapá
Calçoene	1.392	960	1.004	1.205	2.112	1.284	1.489	1.349	11.18	7.83	5.93	9.35	15.85	10.10	11.48	Local Center (5)	Macapá
Pedra Branca do Amaparí	443	442	1.051	703	510	995	1.754	843	3.56	3.61	6.21	5.45	3.83	7.83	13.53	Local Center (5)	Macapá
Serra do Navio	675	466	843	500	425	980	1.692	797	5.42	3.80	4.98	3.88	3.19	7.71	13.05	Local Center (5)	Macapá
Total									**99.47**	**95.52**	**94.03**	**91.24**	**90.12**	**85.64**	**93.95**		
AMAZONAS	98.003	71.650	57.721	80.582	74.826	65.683	73.174	74.520									

States / Municipalities	Malaria cases							Annual average (Malaria cases)	Share of the Municipalities in the States (%)							Managerial centers of the territory [1]	Municípalities of geopolítical and social influence
	**2009**	**2010**	**2011**	**2012**	**2013**	**2014**	**2015**		**2009**	**2010**	**2011**	**2012**	**2013**	**2014**	**2015**		
Manaus	19.372	17.547	16.147	11.610	7.016	7.261	9.623	12.654	19.77	24.49	27.97	14.41	9.38	11.05	13.15	Metropolis (1c)	Manicoré, Tabatinga, Borba, Coari, Eirunepé, Barcelos, Humaitá, Lábrea, Porto Velho, Cruzeiro do Sul, Santarém
Eirunepé	1.973	3.725	3.622	9.042	8.485	5.284	6.239	5.481	2.01	5.20	6.28	11.22	11.34	8.04	8.53	Center of Zone B (4b)	Manaus, Tefé, Coari
São Gabriel da Cachoeira	3.658	9.535	5.098	3.904	5.517	4.531	5.069	5.330	3.73	13.31	8.83	4.84	7.37	6.90	6.93	Local Center (5)	Manaus, Barcelos, Tabatinga
Lábrea	1.891	1.703	1.797	3.958	4.625	7.400	5.147	3.789	1.93	2.38	3.11	4.91	6.18	11.27	7.03	Center of Zone B (4b)	Manaus, Porto Velho, Humaitá
Ipixuna	4.487	515	672	4.011	5.362	2.884	6.702	3.519	4.58	0.72	1.16	4.98	7.17	4.39	9.16	Local Center (5)	Cruzeiro do Sul

States / Municipalities	Malaria cases							Annual average (Malaria cases)	Share of the Municipalities in the States (%)							Managerial centers of the territory [1]	Municípalities of geopolítical and social influence
	2009	2010	2011	2012	2013	2014	2015		2009	2010	2011	2012	2013	2014	2015		
Atalaia do Norte	3.896	3.010	2.467	5.055	3.536	2.870	2.855	3.384	3.98	4.20	4.27	6.27	4.73	4.37	3.90	Local Center (5)	Tabatinga
Coari	6.221	3.270	2.398	2.936	2.551	2.191	2.145	3.102	6.35	4.56	4.15	3.64	3.41	3.34	2.93	Local Center (5)	Manaus, Tefé, Eirunepé
Barcelos	3.100	3.227	2.138	2.376	2.505	3.876	4.211	3.062	3.16	4.50	3.70	2.95	3.35	5.90	5.75	Local Center (5)	Manaus, São Gabriel da Cachoeira
Tefe	5.473	2.194	2.259	3.124	2.898	2.704	2.736	3.055	5.58	3.06	3.91	3.88	3.87	4.12	3.74	Center of sub-region B (3b)	Manaus, Tabatinga, Coari, Sã Paulo de Olivença, Eirunepé, Alvarães, Uarini

States / Municipalities	Malaria cases							Annual average (Malaria cases)	Share of the Municipalities in the States (%)							Managerial centers of the territory [1]	Municípalities of geopolítical and social influence
	2009	2010	2011	2012	2013	2014	2015		2009	2010	2011	2012	2013	2014	2015		
Tabatinga	4.258	2.078	1.291	3.947	3.072	1.580	1.877	2.586	4.34	2.90	2.24	4.90	4.11	2.41	2.57	Center of Zone A (4a)	Manaus, Atalaia do Norte, Benjamin Constant, Santo Antônio do Içá, São Paulo de Olivença, São Gabriel da Cachoeira, Tefé
São Paulo de Olivença	2.441	865	680	4.174	4.186	1.482	2.775	2.372	2.49	1.21	1.18	5.18	5.59	2.26	3.79	Local Center (5)	Manaus, Tefé, Tabatinga
Alvarães	2.787	938	575	1.831	2.498	1.269	1.663	1.652	2.84	1.31	1.00	2.27	3.34	1.93	2.27	Local Center (5)	Tefé
Tapauá	2.959	1.661	1.119	1.412	1.015	1.981	1.361	1.644	3.02	2.32	1.94	1.75	1.36	3.02	1.86	Local Center (5)	Manaus
Benjamin Constant	1.981	957	507	2.873	2.468	715	1.460	1.566	2.02	1.34	0.88	3.57	3.30	1.09	2.00	Local Center (5)	Manaus, Tabatinga

States / Municipalities	Malaria cases							Annual average (Malaria cases)	Share of the Municipalities in the States (%)							Managerial centers of the territory [1]	Municípalities of geopolítical and social influence
	2009	2010	2011	2012	2013	2014	2015		2009	2010	2011	2012	2013	2014	2015		
Guajará	1.707	1.020	874	1.599	2.345	1.691	1.545	1.540	1.74	1.42	1.51	1.98	3.13	2.57	2.11	Local Center (5)	Cruzeiro do Sul
Santo Antônio do Iça	1.673	403	290	1.532	1.294	2.322	2.372	1.412	1.71	0.56	0.50	1.90	1.73	3.54	3.24	Local Center (5)	Manaus, Tabatinga
Uarini	1.536	1.175	685	884	1.439	1.298	1.787	1.258	1.57	1.64	1.19	1.10	1.92	1.98	2.44	Local Center (5)	Manaus, Tefé
Borba	3.210	1.753	2.429	667	112	44	9	1.175	3.28	2.45	4.21	0.83	0.15	0.07	0.01	Local Center (5)	Manaus, Manicoré, Itacoatiara
Humaitá	1.204	1.181	1.834	585	891	1.419	556	1.096	1.23	1.65	3.18	0.73	1.19	2.16	0.76	Local Center (5)	Manaus, Lábrea, Porto Velho
Manicoré	1.628	1.814	1.221	404	253	652	315	898	1.66	2.53	2.12	0.50	0.34	0.99	0.43	Local Center (5)	Manaus, Borba, Porto Velho
Pauini	1.188	336	181	676	1.076	1.518	923	843	1.21	0.47	0.31	0.84	1.44	2.31	1.26	Local Center (5)	Manaus, Lábrea
Itacoatiara	2.395	1.022	271	227	121	118	22	597	2.44	1.43	0.47	0.28	0.16	0.18	0.03	Center of Zone A (4a)	Manaus, Borba, Silves

States / Municipalities	Malaria cases							Annual average (Malaria cases)	Share of the Municipalities in the States (%)							Managerial centers of the territory [1]	Municípalities of geopolítical and social influence
	2009	2010	2011	2012	2013	2014	2015		2009	2010	2011	2012	2013	2014	2015		
									**80.65**	**83.64**	**84.12**	**82.93**	**84.55**	**83.87**	**83.90**		
PARÁ	99.437	132.982	113.874	77.737	24.037	10.593	8.800	66.780									
Anajás	26.073	22.646	17.446	18.824	3.004	1.751	2.476	13.174	26.22	17.03	15.32	24.21	12.50	16.53	28.14	Local Center (5)	Macapá, Breves
Itaituba	6.193	9.933	9.862	11.523	8.970	3.774	1.816	7.439	6.23	7.47	8.66	14.82	37.32	35.63	20.64	Center of sub-region B (3b)	Jacareacanga, Novo Progresso
Oeiras do Para	63	14.531	12.764	5.666	91	4	1	4.731	0.06	10.93	11.21	7.29	0.38	0.04	0.01	Local Center (5)	Cametá
Jacareacanga	5.701	7.552	5.168	4.839	2.516	947	731	3.922	5.73	5.68	4.54	6.22	10.47	8.94	8.31	Local Center (5)	Itaituba
Cametá	25	3.445	13.710	2.449	194	10	1	2.833	0.03	2.59	12.04	3.15	0.81	0.09	0.01	Center of sub-region B (3b)	Oeiras do Pará
Breves	2.546	2.546	6.600	3.707	2.539	832	571	2.763	2.56	1.91	5.80	4.77	10.56	7.85	6.49	Center of sub-region B (3b)	Bagre, Curralinho, Anajás, Portel

States / Municipalities	Malaria cases							Annual average (Malaria cases)	Share of the Municipalities in the States (%)							Managerial centers of the territory [1]	Municípalities of geopolítical and social influence
	2009	2010	2011	2012	2013	2014	2015		2009	2010	2011	2012	2013	2014	2015		
Curralinho	1.800	7.628	4.383	1.714	130	35	25	2.245	1.81	5.74	3.85	2.20	0.54	0.33	0.28	Local Center (5)	Breves
Novo Progresso	3.804	3.553	2.328	2.531	1.635	696	301	2.121	3.83	2.67	2.04	3.26	6.80	6.57	3.42	Local Center (5)	Itaituba
Goianésia do Pará	4.111	5.749	3.017	1.197	206	17	2	2.043	4.13	4.32	2.65	1.54	0.86	0.16	0.02	Local Center (5)	Marabá, Tucuruí
Pacajá	4.409	4.004	2.630	1.348	460	321	177	1.907	4.43	3.01	2.31	1.73	1.91	3.03	2.01	Local Center (5)	Altamira, Tucuruí
Bagre	64	5.408	3.794	1.930	32	7	1	1.605	0.06	4.07	3.33	2.48	0.13	0.07	0.01	Local Center (5)	Breves
Afuá	2.507	1.919	1.076	2.200	652	550	681	1.369	2.52	1.44	0.94	2.83	2.71	5.19	7.74	Local Center (5)	Macapá
Tucuruí	1.892	3.412	1.712	1.549	406	103	162	1.319	1.90	2.57	1.50	1.99	1.69	0.97	1.84	Center of sub-region B (3b)	Pacajá, Altamira, Goianésia do Pará, Jacundá, Marabá
Anapu	893	2.216	3.970	1.583	248	154	24	1.298	0.90	1.67	3.49	2.04	1.03	1.45	0.27	Local Center (5)	Pacajá

States / Municipalities	Malaria cases							Annual average (Malaria cases)	Share of the Municipalities in the States (%)							Managerial centers of the territory [1]	Munícipalities of geopolítical and social influence
	2009	2010	2011	2012	2013	2014	2015		2009	2010	2011	2012	2013	2014	2015		
Altamira	1.863	2.216	2.050	2.089	309	134	32	1.242	1.87	1.67	1.80	2.69	1.29	1.26	0.36	Center of sub-region B (3b)	Manaus, Oriximiná, Anapu, Marabá, Pacajá
Paragominas	4.416	2.501	592	703	56	1	6	1.182	4.44	1.88	0.52	0.90	0.23	0.01	0.07	Center of sub-region B (3b)	Ipixuna do Pará
Ipixuna do Para	4.045	2.316	962	829	26	0	1	1.168	4.07	1.74	0.84	1.07	0.11	0.00	0.01	Local Center (5)	Paragominas
Portel	2.599	1.190	1.868	1.293	753	235	142	1.154	2.61	0.89	1.64	1.66	3.13	2.22	1.61	Local Center (5)	Macapá, Breves
Jacundá	1.120	1.990	550	409	50	6	1	589	1.13	1.50	0.48	0.53	0.21	0.06	0.01	Local Center (5)	Marabá, Tucuruí
Marabá	1.208	1.352	731	256	33	32	16	518	1.21	1.02	0.64	0.33	0.14	0.30	0.18	Regional Capital C (2c)	Altamira, Goianésia do Pará, Tucuruí, Jacundá
Cachoeira do Piria	2.418	572	467	32	0	0	0	498	2.43	0.43	0.41	0.04	0.00	0.00	0.00	Local Center (5)	-

States / Municipalities	Malaria cases							Annual average (Malaria cases)	Share of the Municipalities in the States (%)							Managerial centers of the territory [1]	Municípalities of geopolítical and social influence
	**2009**	**2010**	**2011**	**2012**	**2013**	**2014**	**2015**		**2009**	**2010**	**2011**	**2012**	**2013**	**2014**	**2015**		
Oriximiná	2.661	493	70	13	37	29	13	474	2.68	0.37	0.06	0.02	0.15	0.27	0.15	Local Center (5)	Altamira
Nova Esperança do Piria	1.360	949	139	7	2	0	0	351	1.37	0.71	0.12	0.01	0.01	0.00	0.00	Local Center (5)	-
Total									**82.23**	**81.30**	**84.21**	**85.79**	**92.98**	**90.98**	**81.59**		
RONDÔNIA	38.071	40.622	27.453	21.624	12.892	9.118	6.381	22.309									
Porto Velho	18.786	21.565	15.244	14.265	8.164	5.890	3.121	12.434	49.34	53.09	55.53	65.97	63.33	64.60	48.91	Regional Capital B (2b)	Manaus, Cruzeiro do Sul, Lábrea, Humaitá, Manicoré, Candeias do Jamari, Cujubim, Ariquemes, Alto Paraíso, Buritis
Candeias do Jamari	2.918	3.136	3.210	2.161	1.277	1.186	987	2.125	7.66	7.72	11.69	9.99	9.91	13.01	15.47	Local Center (5)	Porto Velho

States / Municipalities	Malaria cases 2009	2010	2011	2012	2013	2014	2015	Annual average (Malaria cases)	Share of the Municipalities in the States (%) 2009	2010	2011	2012	2013	2014	2015	Managerial centers of the territory [1]	Municípalities of geopolítical and social influence
Machadinho D'Oeste	2.746	2.641	1.866	1.079	599	258	484	1.382	7.21	6.50	6.80	4.99	4.65	2.83	7.59	Local Center (5)	Ariquemes
Cujubim	2.164	1.528	1.081	440	389	209	113	846	5.68	3.76	3.94	2.03	3.02	2.29	1.77	Local Center (5)	Porto Velho, Ariquemes
Ariquemes	1.700	1.905	1.132	516	333	202	120	844	4.47	4.69	4.12	2.39	2.58	2.22	1.88	Center of sub-region B (3b)	Porto Velho, Buritis, Alto Paraíso, Cujubum, Machadinho D'Oeste
Buritis	965	931	536	313	200	69	292	472	2.53	2.29	1.95	1.45	1.55	0.76	4.58	Local Center (5)	Porto Velho, Ariquemes
Alto Paraiso	1.104	1.107	586	140	29	61	52	440	2.90	2.73	2.13	0.65	0.22	0.67	0.81	Local Center (5)	Porto Velho, Ariquemes
Total									**79.8 1**	**80.7 8**	**86.1 7**	**87.4 7**	**85.2 5**	**86.3 7**	**81.0 1**		
RORAIMA	13.29 0	18.702	11.706	5.863	4.772	5.614	6.131	9.440									

States / Municipalities	Malaria cases							Annual average (Malaria cases)	Share of the Municipalities in the States (%)							Managerial centers of the territory [1]	Municípalities of geopolítical and social influence
	2009	2010	2011	2012	2013	2014	2015		2009	2010	2011	2012	2013	2014	2015		
MATO GROSSO	2.412	1.585	1.133	692	713	673	837	1.149									
MARANHÃO	3.714	2.028	2.157	941	529	588	162	1.446									
TOCANTINS	12	9	6	1	6	1	0	5									

Note: (1c) Metropolis with an extensive area of direct influence with a population of over 2 million inhabitants; (2b) regional areas of influence referred to as destination for a set of activities by a large number of municipalities, with a median of 435,000 inhabitants and 406 relationships; (2c) with medians of 250,000 inhabitants and 162 relationships; (3a) their relationships are generally only with the three national metropolis, with medians of 95 thousand inhabitants and 112 relationships; (3b) with medians of 71,000 inhabitants and 71 relationships; (4a), with activities restricted to their immediate area, perform elementary management functions with medians of 45,000 inhabitants and 49 relationships; (4b) with medians of 23,000 inhabitants and 16 relationships; (5) cities whose centrality and performance do not exceed the boundaries of their municipality, serving only their inhabitants, have a population of less than 10,000 inhabitants.

[1] Management centers of the territory: size of the region of influence, in terms of the resident population of the municipalities for which the center represents the focus and the relationship, indicated by the frequency of mentions as the destination for the municipalities.
